# Menahydroquinone-4 may play a key role in regulating CCL5 expression induced by epidermal growth factor receptor inhibitors

**DOI:** 10.1038/s41598-023-49627-8

**Published:** 2023-12-13

**Authors:** Shotaro Goto, Shuichi Setoguchi, Daisuke Watase, Hirofumi Yamakawa, Ayano Yamada, Mitsuhisa Koga, Koichi Matsuo, Kazuhisa Matsunaga, Yoshiharu Karube, Jiro Takata

**Affiliations:** 1 Faculty of Pharmaceutical Sciences, Fukuoka University, 8-19-1 Nanakuma, Jonan-Ku, Fukuoka, 814-0180 Japan; 2 Radioisotope Center, Fukuoka University, Fukuoka, 814-0180 Japan

**Keywords:** Biochemistry, Cancer, Drug discovery, Immunology, Health care

## Abstract

Epidermal growth factor receptor (EGFR) inhibitors frequently cause severe skin rash as a side effect, which is a critical burden for patients who continuously receive drug treatments. Several recent clinical trials have shown that vitamin K is effective against these side effects; however, the underlying mechanisms remain unclear. EGFR inhibitors induce C–C motif chemokine ligand 5 (CCL5) in dermopathy. We hypothesized that menahydroquinone-4 (MKH), the active form of menaquinone-4 (MK-4, vitamin K_2(20)_), supplied by biosynthesis or external delivery, is essential for the suppressive effect on CCL5. The aim of this study was to explore the underlying mechanisms governing the relieving effects of MKH against skin rashes caused by EGFR inhibitors. The responses generated by EGFR inhibitors and the effect of MKH derivatives (two ester derivatives and MK-4) on them were evaluated using human skin cell lines (HaCaT and HSC-1). EGFR inhibitors downregulated UbiA prenyltransferase domain-containing protein-1 (UBIAD1, MKH synthetase) expression and MKH biosynthesis. Knockdown of UBIAD1 or γ-glutamyl carboxylase and treatment with warfarin upregulated CCL5 expression. MKH derivatives suppressed the CCL5 expression induced by EGFR inhibitors. Our data strongly suggest that MKH is involved in suppressing CCL5 expression and alleviating the skin damage caused by EGFR inhibitors.

## Introduction

In recent years, epidermal growth factor receptor (EGFR)-targeted agents have primarily been used for cancer treatment. EGFR inhibitors consist of tyrosine kinase inhibitors (TKIs) and monoclonal antibodies, which are employed for the treatment of colorectal cancer and lung cancer, respectively. However, although EGFR inhibitors generate strong anti-cancer effects, they often induce skin rashes, which substantially affect quality of life and impact medication adherence^1–4^. The occurrence of skin rashes is highly correlated with the efficacy of EGFR inhibitors^1,5,6^. Nevertheless, severe skin rashes can cause both physical and psychological distress, prompting dose reduction or discontinuation of anti-cancer therapy^5,6^. Therefore, meticulous management of such skin rashes becomes imperative.

To date, no prophylactic methods for EGFR inhibitor-induced skin rashes have been established. The general approach to skin rash is symptomatic treatment using topical steroids and antibiotic agents^7^; however, more efficacious treatment strategies are required as certain patients continue suffering from severe skin rashes^8^. Furthermore, increased focus on drug resistance and the side effects associated with long-term drug use is warranted.

Recently, several clinical trials have reported that external skin preparations for vitamin K_1_ (phylloquinone, PK) are effective against EGFR inhibitor-induced skin rashes^9–11^. However, the underlying mechanisms remain unelucidated.

**Figure 1:**
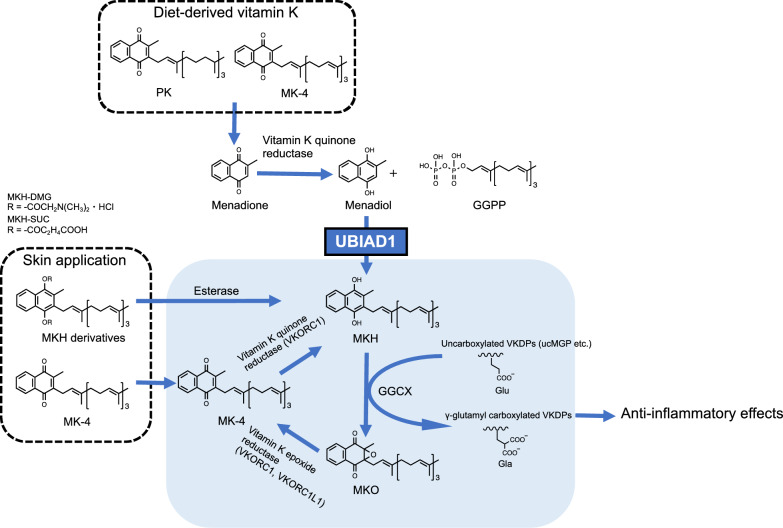
Schematic illustration of the vitamin K cycle associated with the anti-inflammatory effects and concept of the intracellular biosynthesis or delivery system of menahydroquinone-4. *PK* phylloquinone, *MKH* menahydroquinone-4, *MK-4* menaquinone-4, *MKO* menaquinone-4 epoxide, *VK* vitamin K, *VKDP* vitamin K-dependent protein, *GGCX* γ-glutamyl carboxylase, *VKORC1* vitamin K epoxide reductase complex subunit 1, *VKORC1L1* vitamin K epoxide reductase complex subunit 1-like 1, *DTT* dithiothreitol, *GGPP* geranyl geranyl pyrophosphate, *UBIAD1* UbiA prenyltransferase domain-containing protein-1, *ucMGP* uncarboxylated matrix Gla protein, *Glu* glutamate, *Gla* γ-carboxyglutamate, *MKH-DMG* menahydroquinone-4 1,4-bis-*N,N*-dimethylglycinate hydrochloride, *MKH-SUC* menahydroquinone-4 1,4-bis-hemisuccinate.

Both PK and Vitamin K_2_ (menaquinone-4, MK-4) obtained from the diet are converted into vitamin K_3_ (menadione) in the gastrointestinal tract and then reduced to menadiol (Fig. 1). Subsequently, menadiol is prenylated to menahydroquinone-4 (MKH) via UbiA prenyltransferase domain-containing protein 1 (UBIAD1)^12–14^. MKH is the fully reduced and active form of MK-4 and functions as a cofactor for γ-glutamyl carboxylase (GGCX), an enzyme that converts γ-glutamate (Glu) residues to γ-carboxyglutamate (Gla) residues during the post-translational modification of vitamin K-dependent proteins (VKDPs)^15,16^. MKH promotes the conversion of Glu to Gla in VKDPs via GGCX, after which MKH is stoichiometrically converted to menaquinone-4 2,3-epoxide (MKO). MKO is subsequently regenerated to MK-4 via the action of vitamin K epoxide reductase complex subunit 1 (VKORC1) or vitamin K epoxide reductase complex subunit 1-like 1 (VKORC1L1). This series of redox reactions is termed the vitamin K cycle. Vitamin K is involved in various biological functions, including blood clotting, bone formation, and anti-inflammatory effects, through the conversion of Glu to Gla in VKDPs^17^. Therefore, we hypothesized that adequate MKH delivery to the skin may suppress EGFR inhibitor-induced skin rashes.

We previously reported that ester-type derivatives (Fig. 1) menahydroquinone-4 1,4-bis-*N,N*-dimethylglycinate hydrochloride (MKH-DMG) and menahydroquinone-4 1,4-bis-hemisuccinate (MKH-SUC) deliver MKH to skin cells more efficiently than MK-4^18^. Furthermore, MKH derivatives are more suitable for skin use because of their higher photostability than MK-4.

Herein, using a normal human keratinocyte cell line (HaCaT cells) and human skin squamous cell carcinoma cell line (HSC-1), we aimed to explore the mechanisms underlying the relieving effects of MKH against skin rashes caused by EGFR inhibitors. In the present study, a novel strategy for managing skin toxicity caused by EGFR inhibitors has been delineated.

## Results

### EGFR inhibitors downregulated UBIAD1 expression and upregulated CCL5 expression

**Figure 2:**
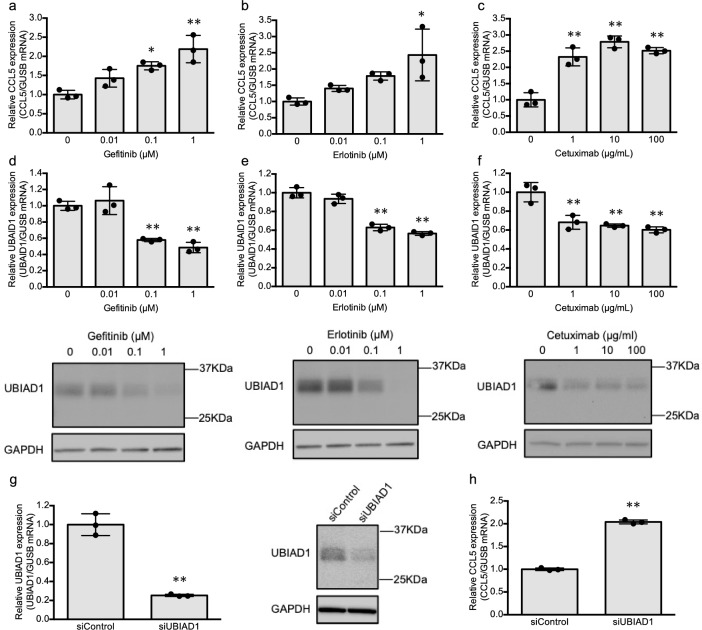
Effects of EGFR inhibitors on CCL5 and UBIAD1 expression in HaCaT cells. HaCaT cells were assessed using real-time quantitative RT-PCR and western blot analysis after incubation for 24 h with gefitinib (**a**, **d**), erlotinib (**b**, **e**), cetuximab (**c**, **f**), siUBIAD1 under indicated conditions. Relative mRNA expression levels of CCL5 and UBIAD1 were normalized to GUSB mRNA expression. UBIAD1 protein expression was normalized to GAPDH protein expression. The whole blot image can be found in Supplementary Fig. 3. The values represent means ± standard deviation (n = 3). *p < 0.05, **p < 0.01 compared to the control (0 µM) (**a**–**f**) or siControl (**g**, **h**). *CCL5* chemokine ligand-5, *EGFR* epidermal growth factor receptor, *GUSB* β-glucuronidase, *RT-PCR* reverse transcription-polymerase chain reaction, *UBIAD1* UbiA prenyltransferase domain-containing 1, *GAPDH* glyceraldehyde-3-phosphate dehydrogenase, *EGFR* epidermal growth factor receptor.

CCL5 is upregulated at the skin rash sites in patients administered EGFR inhibitors, which generally attracts leukocytes to inflammation sites^19,20^. Therefore, an increase in CCL5 levels can lead to rash exacerbation. To confirm whether EGFR inhibitors upregulated CCL5 expression in vitro, we evaluated CCL5 mRNA expression in HaCaT cells treated with gefitinib, erlotinib, and cetuximab. CCL5 mRNA expression increased with gefitinib (0.01, 0.1, and 1.0 µM), erlotinib (0.01, 0.1, and 1.0 µM), and cetuximab (1, 10, and 100 µg/mL) treatment in a concentration-dependent manner (Fig. 2a–c), as previously reported^21–23^. Therefore, this model conclusively demonstrated the phenomenon of EGFR inhibitor-induced skin rashes.

To confirm the effect of EGFR inhibitors on the MKH biosynthetic pathway, the effects of EGFR inhibitors on UBIAD1 mRNA and protein expression were evaluated. UBIAD1 mRNA and protein levels decreased with EGFR inhibitor treatment in a concentration-dependent manner (Fig. 2d–f). A similar result was obtained in HSC-1 cells following gefitinib treatment (Supplementary Fig. 1a) and suggests that EGFR inhibitors downregulate UBIAD1 expression, which concurrently upregulates CCL5 expression in skin cells. Furthermore, to elucidate the correlation between UBIAD1 and CCL5, the effects of UBIAD1 knockdown on CCL5 expression were evaluated. siUBIAD1 treatment reduced UBIAD1 mRNA and protein levels in HaCaT cells (Fig. 2g) but upregulated CCL5 mRNA expression (Fig. 2h), implying that UBIAD1 regulates CCL5 expression.

### EGFR inhibitors suppressed MKH biosynthesis

**Figure 3:**
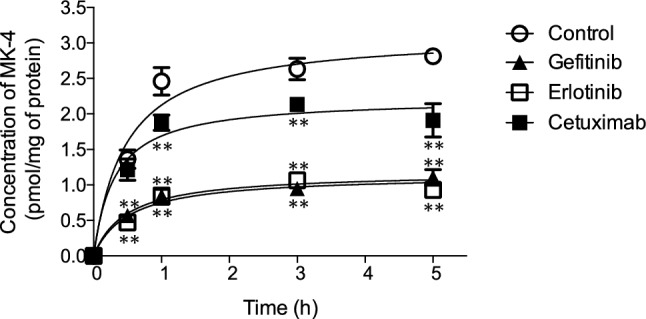
Effect of EGFR inhibitors on MK-4 conversion activity in S9 fractions of HaCaT cells. HaCaT cells cultured with 1 µM gefitinib, 1 µM erlotinib, or 100 µg/mL cetuximab for 24 h were harvested and S9 fractions were prepared. Total protein concentration of S9 fractions was normalized in PBS and the suspension was incubated with 100 μM menadione, 100 μM GGPP, and 1 mM DTT for 5 h at 37 °C. Concentrations of MK-4 synthesized over time were determined using LC–MS/MS. Values represent means ± standard deviation (n = 3). **p < 0.01 compared to the control at the same time point. *DTT* dithiothreitol, *EGFR* epidermal growth factor receptor, *GGPP* geranylgeranyl pyrophosphate, *LC–MS/MS* liquid chromatography with tandem mass spectrometry, *MK-4* menaquinone-4.

As EGFR inhibitors downregulate UBIAD1 expression in HaCaT cells, we hypothesized that MKH biosynthesis would also be hindered. To assess the effect of EGFR inhibitors on MKH biosynthesis, we measured MKH concentration in the S9 fraction of HaCaT cells cultured with gefitinib (1 µM), erlotinib (1 µM), or cetuximab (100 µg/mL). Enzymatic reactions were initiated by adding menadione (100 µM), GGPP (100 µM), and DTT (1 mM) into S9 fraction-containing tubes. The MKH produced was easily oxidized to MK-4 during extraction. Consequently, MK-4 concentration was measured using LC–MS/MS and regarded as the MKH concentration. In each group, MK-4 concentrations increased in a time-dependent manner and plateaued after 1 h. S9 fractions cultured with EGFR inhibitors showed significantly lower MK-4 levels than the control (Fig. 3). Gefitinib and erlotinib equally suppressed MKH production, to a greater extent than cetuximab.

To reaffirm the obtained result, we showed a concentration-dependent increase in uncarboxylated matrix Gla protein (ucMGP), an indicator of decreased conversion of Glu to Gla in VKDPs, in gefitinib-treated HaCaT cells (Supplementary Fig. 2a). Of note, no significant effect of gefitinib on VKORC1, VKORC1L1 and GGCX mRNA expression were observed (Supplementary Fig. 2b–d). As MKH promotes the conversion of Glu to Gla in VKDPs if GGCX is functional, an increased ucMGP content implies the suppression of the biosynthesis of MKH. Altogether, these results indicate that EGFR inhibitors suppress the biosynthesis of MKH by downregulating UBIAD1 expression and affecting the conversion of Glu to Gla in VKDPs, which could lead to upregulated CCL5 mRNA expression.

### Suppression of MKH levels and Glu to Gla conversion in VKDPs lead to the upregulation of CCL5 expression

**Figure 4:**
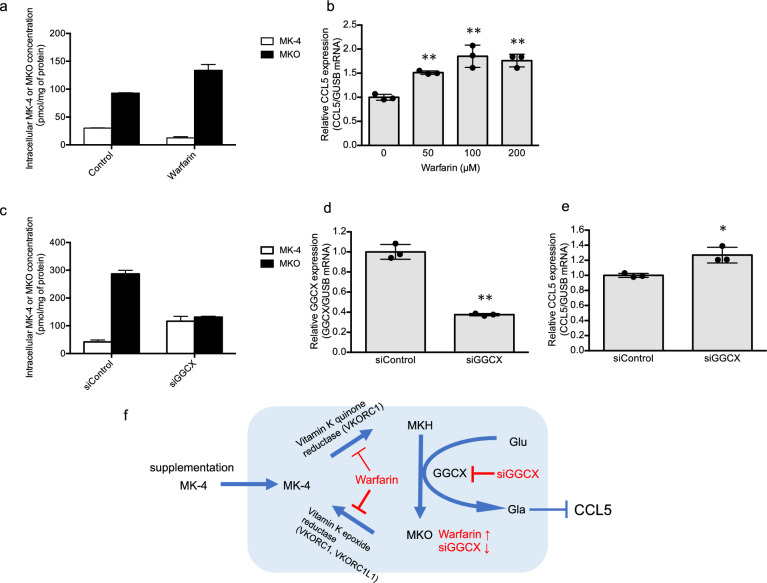
Effect of warfarin and siGGCX on CCL5 mRNA expression in HaCaT cells. HaCaT cells were treated with various concentrations of warfarin (**a**, **b**) or siGGCX (**c**–**e**) for 24 h and assessed for measurement of MK-4 and MKO concentration after 3 µM (**a**) or 10 µM (**c**) MK-4 treatment and CCL5 (**b**, **e**) or GGCX (**d**) mRNA expression using real-time quantitative RT-PCR. Relative mRNA expression levels of CCL5 and GGCX were normalized to GUSB mRNA expression. Concentrations of MK-4 and MKO were determined using LC–MS/MS. Values represent means ± standard deviation (n = 3). *p < 0.05, **p < 0.05, compared to the control (0 µM or siControl). (**f**) Graphic summary depicting changes in intracellular MKO levels following warfarin or siGGCX treatment and the modulation of CCL5 expression by Gla proteins. *CCL5* chemokine ligand-5, *GGCX* γ-glutamyl carboxylase, *GUSB* β-glucuronidase, *MK-4* menaquinone-4, *MKO* menaquinone-4 2,3-epoxide, *LC–MS/MS* liquid chromatography with tandem mass spectrometry; RT-PCR, reverse transcription-polymerase chain reaction.

The anticoagulant warfarin inhibits the activities of VKORC1 and VKORC1L1^24,25^. These enzymes catalyze the reduction of MKO and MK-4 to MKH, which are required for the Glu to Gla conversion in VKDPs. Consequently, the inhibition of VKORC1 and VKORC1L1 restricts the availability of MKH, leading to the suppression of Glu to Gla conversion in VKDPs. To confirm whether reduced levels of MKH affect CCL5 expression, we evaluated the effect of warfarin on CCL5 mRNA expression in HaCaT cells. The inhibition of the activity of VKORC1 was confirmed by the increased intracellular MKO concentration via the administration of 3 µM MK-4 following treatment with 200 µM warfarin (Fig. 4a). Furthermore, treatment with warfarin (50, 100, and 200 µM) upregulated CCL5 mRNA expression in a dose-dependent manner (Fig. 4b). Next, to investigate whether conversion of Glu to Gla in VKDPs affects CCL5 expression, we evaluated the effects of GGCX knockdown on CCL5 mRNA expression. The decrease in GGCX activity was confirmed by the decreased intracellular MKO concentration via the administration of 10 µM MK-4 following siGGCX treatment (Fig. 4c). Treatment with siGGCX suppressed GGCX mRNA expression and upregulated CCL5 mRNA expression (Fig. 4d,e). These results indicate that MKH functions as a cofactor of GGCX and promotes the conversion of Glu to Gla in VKDPs, thereby regulating CCL5 expression. Graphic summary depicting changes in intracellular MKO levels following warfarin or siGGCX treatment and the modulation of CCL5 expression by Gla proteins are presented in Fig. 4f.

### MK-4 and MKH derivatives suppressed CCL5 expression

**Figure 5:**
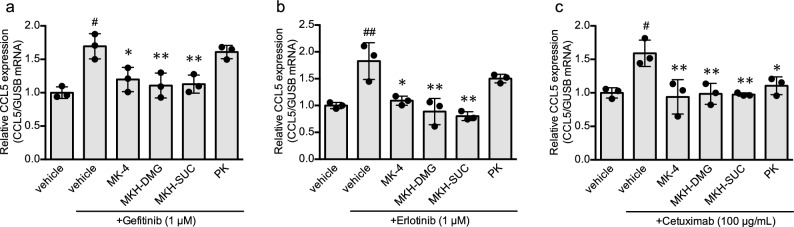
Effect of MK-4, PK, and MKH derivatives on CCL5 expression induced by EGFR inhibitors in HaCaT cells. HaCaT cells were pretreated with 3 µM MK-4, MKH-DMG, MKH-SUC, or PK for 24 h and then treated with 1 µM gefitinib (**a**), 1 µM erlotinib (**b**), or 100 µg/mL cetuximab (**c**) for 24 h. Relative mRNA expression levels of CCL5 was normalized to GUSB mRNA expression. The values represent means ± standard deviation (n = 3). ^#^p < 0.05, ^##^p < 0.01 compared to the vehicle. *p < 0.05, **p < 0.01 compared to those treated with EGFR inhibitors only. *CCL5* chemokine ligand-5, *EGFR* epidermal growth factor receptor, *MK-4* menaquinone-4, *MKH-DMG* menahydroquinone-4 1,4-bis-*N,N*-dimethylglycinate hydrochloride, *MKH-SUC* menahydroquinone-4 1,4-bis-hemisuccinate, *PK* phylloquinone.

We hypothesized that efficient MKH delivery could regulate CCL5 expression; furthermore, we evaluated the effects of MK-4, PK, and MKH derivatives (MKH-DMG and MKH-SUC) on CCL5 mRNA expression in HaCaT cells, which was upregulated by EGFR inhibitors. Pretreatment with 3 µM MK-4, MKH-DMG, and MKH-SUC showed a significant suppressive effect on CCL5 mRNA expression, which was initially upregulated by gefitinib (1 µM), erlotinib (1 µM), and cetuximab (100 µg/mL) (Fig. 5a–c). In contrast, PK significantly downregulated only EGFR monoclonal antibody (cetuximab)-induced CCL5 mRNA expression (Fig. 5c). The effect of MKH derivatives was similar in gefitinib-treated HSC-1 cells (Supplementary Fig. 1b). These results indicate that bolstering intracellular MKH delivery is crucial for regulating CCL5 expression in skin cells.

## Discussion

EGFR plays a pivotal role in epidermal development and physiology^26^. Blocking EGFR signaling in skin cells downregulates several pathways, including the mitogen-activated protein kinase, phosphatidylinositol-3-kinase, and stress-activated protein kinase pathways. EGFR inhibition causes cell growth arrest and apoptosis, which interfere with the structural development of the skin. It also induces CCL2, CCL3, CCL5, CCL18, CXC motif chemokine ligand (CXCL) 9, CXCL10, XCL1, fractalkine (CX3CL1), and CXC chemokine receptor type 4 production^21–23^. These chemokines promote the recruitment and activation of leukocytes, such as neutrophils, monocytes, and lymphocytes, at the skin rash sites. Consequently, the barrier function of the skin is impaired, exacerbating inflammation^21^.

Recently, vitamin K has emerged as a novel therapeutic candidate for EGFR inhibitor-induced skin rashes. Menadione activates ErbB, a member of the TK receptor family that also includes EGFR, through the phosphatase-inhibitory effect of menadione^27^. Perez-Soler and Ling disclosed a patent for a method to activate EGFR in the skin through topical vitamin K treatment to protect the skin from the systemic effects of EGFR inhibitors ^28^. In this patent, the authors reported that 100 μM menadione is the strongest EGFR activator, 1000 μM PK generates 1/10th of the effect of menadione, and MK-4 is ineffectual against human squamous cell carcinoma cells (A431). They subsequently published a research paper based on this patent in Clinical Cancer Research^29^, but it was retracted in 2013. Clinical trials using menadione and PK have been conducted for the prevention and treatment of skin rashes caused by cetuximab^30,31^. Menadione was shown to be ineffective in treating cetuximab-induced skin rashes; however, the efficacy of PK remains an issue of contention. A recent clinical trial (EVITA) showed that the topical application of PK is effective in women^10,32^. There are no clinical trials on the efficacy of menadione and PK in treating EGFR-tyrosine kinase inhibitor (TKI)-related skin rashes. Additionally, the mechanism of action of vitamin K on EGFR inhibitor-induced skin rashes remains to be elucidated.

Dietary PK is converted to menadione in the body, which is then converted to MKH via UBIAD1^12–14^. The findings of our study suggest that patients receiving chronic treatment with EGFR inhibitors have low MKH biosynthesis ability owing to UBIAD1 suppression. Gefitinib and erlotinib reportedly prolong the prothrombin time-international normalized ratio in patients undergoing warfarin treatment; however, the underlying mechanism remains unascertained^33,34^. The suppression of MKH biosynthesis by EGFR inhibitors could be a plausible cause.

Our data also showed that UBIAD1 knockdown, as well as warfarin and siGGCX treatment, upregulated CCL5 mRNA expression, suggesting that MKH delivery and conversion of Glu to Gla in VKDPs are essential for regulating CCL5 expression. VKDPs that exhibit anti-inflammatory effects include protein S, growth-arrest specific gene 6 (Gas6) and Gla-Rich Protein (GRP).Protein S and Gas6 significantly inhibited the expression of pro-inflammatory cytokines such as TNF-α, IL-6, and IL-1β in macrophages following LPS stimulation^35^. GRP suppressed the expression levels of inflammatory markers, including cyclooxygenase 2 (COX2) and matrix metallopeptidase 13 (MMP13), in chondrocytes derived from osteoarthritis and synovial cell lines^36^. These VKDPs may be involved in the regulation of CCL5 expression; however, further studies are needed to clarify this.

For these reasons, we anticipated that efficient MKH delivery would inhibit CCL5 expression. Our data showed that MK-4 and MKH derivatives suppressed CCL5 expression induced by EGFR inhibitors to a greater degree than PK. The ineffectiveness of PK may be attributed to the reduced expression of UBIAD1 caused by EGFR inhibitors, as UBIAD1 plays a crucial role in converting PK to MKH. As a result, the full potential of PK cannot be realized. Furthermore, it is also conceivable that PK exhibits lower cellular uptake and has lower bioavailability than MK-4, contributing to its ineffectiveness on CCL5 expression^18,37^.

CCL5 is an inflammatory chemokine released in the late inflammatory phase that mobilizes leukocytes to inflammatory sites and is involved in maintaining inflammation^20^. Its levels are increased in human skin tissue treated with EGFR inhibitors^19^; a similar phenomenon has been reported in mouse skin tissue^22,38^. In vitro treatment of human skin cells with EGFR inhibitors has been reported to increase CCL5 levels^21–23^. Promotion of CCL5 expression is expected to contribute to the exacerbation of EGFR inhibitor-induced skin rashes. Conversely, CCL5 is also associated with cancer progression and metastasis by promoting the survival, proliferation, and infiltration of tumor cells^38^. Therefore, enhancing the rate of MKH delivery to the skin may also prove efficient in preventing skin cancer metastasis.

Our findings reveal that MKH derivatives produce a CCL5-suppressive effect that is equal to or greater than that exhibited by MK-4. However, MK-4 is easily photodegraded and possesses phototoxic properties, leading to the prohibition of the conventional usage of vitamin K in cosmetics within Europe^39^. Consequently, the effects of light on MK-4 are unavoidable when MK-4 is used as a topical skin treatment. In contrast, our previous research has demonstrated that MKH derivatives are not easily degraded and do not exhibit phototoxicity^18^. Furthermore, MKH derivatives can deliver MKH to cells, even after irradiation with sunlight. Hence, it can be concluded that MKH derivatives are more suitable for topical skin applications than MK-4.

In conclusion, patients using EGFR inhibitors may have decreased MKH biosynthesis ability and increased CCL5 expression together with reduced UBIAD1 expression. MKH treatment via MK-4 and MKH derivatives may contribute to the suppression of CCL5 expression and alleviation of skin damage caused by EGFR inhibitors. Furthermore, MKH derivatives are less sensitive to light and may be more useful as topical skin preparations than MK-4. A limitation of our study is the use of a monolayer culture system, which cannot completely replicate the biological environment. Future analyses using in vivo models are required to obtain more definitive results. We strongly anticipate that our findings will contribute to elucidating the onset and alleviation mechanism of skin rashes.

## Methods

### Chemicals

EGFR-TKIs (gefitinib and erlotinib), PK, and warfarin were purchased from FUJIFILM Wako Pure Chemical Corporation (Osaka, Japan). EGFR monoclonal antibody (cetuximab; Erbitux) was purchased from Merck Inc. (Milan, Italy). MK-4 was purchased from Seebio Biotech Inc. (Shanghai, China). MKH-DMG and MKH-SUC were synthesized using previously reported methods^18^.

### Cell culture

HaCaT cells obtained from CLS Cell Lines Service GmbH (Eppelheim, Germany) and HSC-1 cells obtained from the Japanese Collection of Research Bioresources Cell Bank were grown in high glucose-containing Dulbecco’s modified Eagle’s medium (FUJIFILM Wako Pure Chemical) containing 10% fetal bovine serum and 1% penicillin/streptomycin (Thermo Fisher Scientific, Waltham, MA, USA) at 37 °C under a 5% CO_2_ environment.

### Real-time quantitative reverse transcription-polymerase chain reaction (RT-PCR)

HaCaT or HSC-1 cells (5.0 × 10^4^ cells/well) were seeded in 24-well plates and allowed to attach for 24 h. Cells were cultured in a medium containing 3 µM MK-4, PK, or MKH derivatives for 24 h. Thereafter, the medium was replaced with fresh medium containing gefitinib, erlotinib, or cetuximab at the indicated concentrations and incubated for 24 h. Total RNA was extracted from HaCaT or HSC-1 cells using the High Pure RNA Isolation Kit (Roche, Basel, Switzerland) according to the manufacturer’s instructions. Reverse transcription was performed using ReverTra Ace Master Mix (Toyobo, Osaka, Japan) with a thermal cycler system (Bio-Rad, Hercules, CA, USA), following the manufacturer’s instructions. RT-PCR was performed using the LightCycler 480 System (Roche) on samples with a final volume of 20 µL containing cDNA (25 µg/µL), RT-PCR LightCycler 480 SYBR Green I Master Mix (10 µL) (Roche), and primers. The conditions for the PCR were as follows: 1 cycle of 95 °C (5 min), followed by 50 cycles of 10 s at 95 °C, 10 s at 60 °C, and 10 s at 72 °C. After completion of the last cycle of PCR, the melting curve analysis was performed at one cycle of 95 °C for 5 s, 60 s at 65 °C, and continuous heating at 0.11 °C/s to 97 °C. The primers used were as follows: CCL5 Forward, 5′-TGCCCACATCAAGGAGTATTT-3′; Reverse, 5′-CTTTCGGGTGACAAAGACG-3′; UBIAD1 Forward, 5′-CACTTGGCTCTTATCTACTTTGGAG-3′; Reverse, 5′-GTCTCCCAGAGCCACGTACT-3′; GGCX Forward, 5′-CTGGCTCTTCAGTCCCTTCA-3′; Reverse, 5′-CCAGCTGAGAGGTCAAGCA-3′; β-glucuronidase (GUSB) Forward, 5′-CGCCCTGCCTATCTGTATTC-3′; Reverse, 5′-TCCCCACAGGGAGTGTGTAG-3′; VKORC1 Forward, 5′-GGTCTCAAGCAATCTGTCTGC-3′; Reverse, 5′-AGCAGCATCAGGACAGAGG-3′; VKORC1L1 Forward, 5′-CTGCTCTCCATCTACGCCTAC-3′; Reverse, 5′-CACAGCGCTTGCTGTCAT-3′. mRNA expression was normalized using GUSB as an internal standard.

### Western blot analysis

HaCaT cells (2.0 × 10^5^ cells/well) were seeded on 6-well plates and allowed to attach for 48 h. The medium was replaced with fresh medium containing gefitinib, erlotinib, or cetuximab at the indicated concentrations and incubated for 24 h. HaCaT cells were washed with cold phosphate-buffered saline (PBS) and harvested with a protease inhibitor cocktail (Nacalai Tesque, Inc., Kyoto, Japan) containing radioimmunoprecipitation assay buffer. The lysates were centrifuged at 10,000×*g* for 10 min at 4 °C, and the supernatant protein concentration was determined using a bicinchoninic acid protein (BCA) assay kit (Thermo Fisher Scientific). Samples (20 μg of total protein) were mixed with a sample buffer solution containing a reducing reagent (6 ×) for sodium dodecyl sulfate–polyacrylamide gel electrophoresis (SDS-PAGE) (Nacalai Tesque) and separated using 15% SDS-PAGE (FUJIFILM Wako Pure Chemical). Thereafter, the proteins were transferred onto a polyvinylidene difluoride membrane (Bio-Rad). After blocking with Blocking One buffer (Nacalai Tesque), the membranes were incubated with human Anti-UBIAD1 (TERE1 H-8) antibody (1:1000; #sc-377013; Santa Cruz Biotechnology Inc., Dallas, TX, USA) or human anti- glyceraldehyde-3-phosphate dehydrogenase (GAPDH) antibody (1:10,000; #G8795; Sigma-Aldrich, St. Louis, MO, USA) for 1 h, followed by incubation with peroxidase-conjugated anti-mouse secondary antibody (1:20,000; Cell Signaling Technology, Danvers, MA, USA) for 1 h. Signals were visualized using ImmunoStar LD (FUJIFILM Wako Pure Chemical). The UBIAD1 band size was observed at 25–37 kDa, and the GAPDH band size was observed at 37 kDa.

### siRNA transfection experiments

HaCaT cells (5.0 × 10^4^ cells/well) were seeded in 24-well plates and allowed to attach for 48 h before siRNA transfection. Cells were then transfected with 15 pmol/well siControl, siUBIAD1, or siGGCX (Sigma-Aldrich) for 24 h using Lipofectamine 2000 reagent (Thermo Fisher Scientific) in Opti-MEM (Thermo Fisher Scientific), following the manufacturer’s protocol.

### MKH biosynthesis activity of UBIAD1

MKH biosynthesis ability was assessed using a previously reported method^12^. Briefly, HaCaT cells (5.0 × 10^5^ cells/well) were seeded in a 10-cm dish and allowed to attach for 72 h. The medium was replaced with fresh medium containing gefitinib, erlotinib, or cetuximab and incubated for 24 h. Cells were washed with cold PBS and collected in 1 mL of PBS using a cell scraper. After ultrasonic homogenization, cells were centrifuged at 10,000×*g* for 1 h at 4 °C, and S9 fractions were obtained. MK-4 generation reactions were initiated by mixing 1 mM dithiothreitol (DTT; FUJIFILM Wako Pure Chemical), 100 µM menadione (Sigma-Aldrich), and 100 µM geranylgeranyl pyrophosphate (ammonium salt) (GGPP) (Cayman Chemical Company, Ann Arbor, MI, USA) with S9 fraction solutions adjusted to 760 µg protein/mL in PBS. Solutions were incubated for 5 h at 37 °C, and reactions were stopped with an equal volume of methanol and a triple volume of n-hexane. After vortexing and centrifugation at 1750×*g* for 10 min, the upper layer (n-hexane) was collected and evaporated under N_2_ gas. The residue was reconstituted with methanol (50 μL) and analyzed using liquid chromatography with tandem mass spectrometry (LC–MS/MS) as reported previously^18^. Identification and quantitation were based on MS/MS-multiple reaction monitoring mode using transition ions as follows: *m/z* 445 → 187 for the (M + H)^+^ MK-4 adduct, and *m*/*z* 461 → 81 for the [M + H]^+^ MKO adduct.

### Determination of intracellular MKO and MK-4 levels after treatment with warfarin and siGGCX

HaCaT cells (5.0 × 10^4^ cells/well) were seeded in 24-well plates and allowed to attach for 48 h before warfarin (50, 100, or 200 µM) treatment or siGGCX transfection for 24 h. Subsequently, the medium was replaced with a medium containing MK-4 (3 or 10 µM) and cultured for an additional 24 h. The medium was removed, and the cells were washed twice with PBS. The cells were scraped from the wells, collected in PBS (500 µL), and sonicated. The cell homogenates were combined with an equal volume of methanol and triple volumes of n-hexane, vortexed for 2 min, and centrifuged at 1750×*g* for 10 min. The upper layer (n-hexane) was collected and evaporated under N_2_ gas. The residue was reconstituted with methanol (50 μL) and analyzed using LC–MS/MS, as described in “MKH biosynthesis activity of UBIAD1.” Protein concentrations were determined using a BCA protein assay kit.

### Statistical analysis

Statistical significance was determined using unpaired t-test or variance (ANOVA) followed by Dunnett’s post hoc test. Differences were considered statistically significant at p < 0.05. Data were analyzed using GraphPad Prism 6 (GraphPad Software, San Diego, CA, USA).

### Supplementary Information

Supplementary Figure 1.

Supplementary Figure 2.

Supplementary Figure 3.

## Data Availability

The datasets generated during and/or analysed during the current study are available from the corresponding author on reasonable request.
