# Supplementary material for: Menahydroquinone-4 may play a key role in regulating CCL5 expression induced by epidermal growth factor receptor inhibitors
Source: Sci Rep. 2023 Dec 13;13:22102. doi: 10.1038/s41598-023-49627-8 (PMC10719312; doi:10.1038/s41598-023-49627-8)
Supplement: Supplementary file 1 — Supplementary Figure 1. [file 41598_2023_49627_MOESM1_ESM.docx]

**Supplemental Figure 1. Effects of gefitinib on UBIAD1 expression and MK-4, PK, and MKH derivatives on CCL5 expression induced by gefitinib in HSC-1 cells.**

HSC-1 were cultured with 0.01, 0.1, or 1 µM gefitinib for 24 h. The relative mRNA expression of UBIAD1 was normalized to that of GUSB (a), and the values represent means ± standard deviation (n = 3). ** p < 0.01, compared to the control. HSC-1 cells were pretreated with 3 µM MK-4, MKH-DMG, MKH-SUC, or PK for 24 h and then treated with 1 µM gefitinib for 24 h (b). The values represent means ± standard deviation (n = 3).  ^##^p < 0.01 compared to the vehicle. * p < 0.05, ** p < 0.01 compared to those treated with gefitinib only. CCL5, chemokine ligand-5; UBIAD1, UbiA prenyltransferase domain-containing 1; MK-4, menaquinone-4; MKH-DMG, menahydroquinone-4 1,4-bis-*N,N*-dimethylglycinate hydrochloride; MKH-SUC, menahydroquinone-4 1,4-bis-hemisuccinate; PK, phylloquinone; GUSB, β-glucuronidase
