# Supplementary material for: Menahydroquinone-4 may play a key role in regulating CCL5 expression induced by epidermal growth factor receptor inhibitors
Source: Sci Rep. 2023 Dec 13;13:22102. doi: 10.1038/s41598-023-49627-8 (PMC10719312; doi:10.1038/s41598-023-49627-8)
Supplement: Supplementary file 2 — Supplementary Figure 2. [file 41598_2023_49627_MOESM2_ESM.docx]

**Supplemental Figure 2.** **Effects of gefitinib on ucMGP protein expression and GGCX, VKORC1, and VKORC1L1 mRNA expression in HaCaT cells.** HaCaT cells were cultured with 0.01, 0.1 or 1 µM gefitinib for 24 h. The quantification of ucMGP levels in HaCaT cells was performed using ELISA (a). HaCaT cells were seeded and harvested using the method described in Section 2.4. ucMGP levels in HaCaT cells were measured using a human ucMGP ELISA kit (Cusabio Biotech Co., Wuhan, China) according to the manufacturer’s instructions. Values were adjusted based on protein content, which was determined from the same plates.The relative mRNA expression of GGCX (b), VKORC1 (c), and VKORC1L1 (d) was normalized to that of GUSB, and the values represent means ± standard deviation (n = 3). * p < 0.05, compared to the control. ucMGP, uncarboxylated matrix Gla protein; GUSB, β-glucuronidase; GGCX, γ-glutamyl carboxylase; VKORC1, vitamin K epoxide reductase complex subunit 1; VKORC1L1, vitamin K epoxide reductase complex subunit 1-like 1; ELISA, enzyme-linked immunosorbent assay; n.s., not significant
