# Supplementary material for: Menahydroquinone-4 may play a key role in regulating CCL5 expression induced by epidermal growth factor receptor inhibitors
Source: Sci Rep. 2023 Dec 13;13:22102. doi: 10.1038/s41598-023-49627-8 (PMC10719312; doi:10.1038/s41598-023-49627-8)
Supplement: Supplementary file 3 — Supplementary Figure 3. [file 41598_2023_49627_MOESM3_ESM.docx]

**Supplementary Figure 3. The unprocessed western blots shown Figure 2d, e, f, g images.Cropped areas are highlighted by red box.** HaCaT cells were assessed using western blot analysis after incubation for 24 h with gefitinib (a), erlotinib (a), cetuximab (b), siUBIAD1 (c) under indicated conditions.The bright field image of the UBIADA1 or GAPDH treated membrane and the unprocessed band for the UBIAD1 or GAPDH protein.
